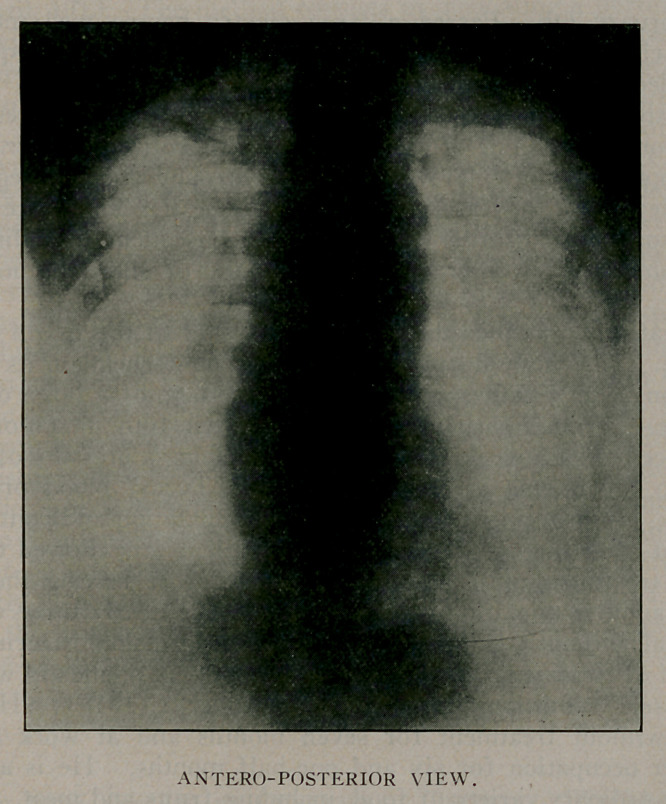# Cardiospasm of Thirty Years’ Duration

**Published:** 1914-02

**Authors:** Eli H. Long

**Affiliations:** Buffalo, N. Y.


### BUFFALO MEDICAL JOURNAL

VOLUME 69 FEBRUARY, 1914 No. 7

### ORIGINAL ARTICLES

*The right is reserved to decline papers not dealing with practical medical and surgical subjects and such as might offend or fail to interest readers. Contributors are solely responsible for opinions, methods of expression and revision of proof.*

### Cardiospasm of Thirty Years' Duration

BY ELI H. LONG, M.D.
Buffalo, N. Y.

THE following case has presented itself for restudy and treatment after an interval of twenty-seven years. With the limited earlier diagnostic methods the condition was believed to be diverticulum of the esophagus. The case was reported in 1886* with the following history:

"C. W., aged 20, cigarmaker by trade, but has not worked at it since December, 1884. Has never been strong. Has been ailing for three years, with symptoms referred to the digestive apparatus. He came under observation February 14, 1885, complaining that he could not retain his food. He was somewhat emaciated and weak, weighed 99 pounds, with pulse 144, and temperature normal.

His food was regurgitated rather than vomited, and he designated a region at the right of the sternum, on a level with the nipple, where he said his food and drink seemed to remain instead of going into the stomach. He would experience pain in this region until regurgitation occurred, which was generally soon after eating. These symptoms were sometimes very bad and at other times not so distressing. His heart sounds and respiratory murmur were normal. Bowels were irregular, appetite fair, and urine normal. He informed me that he had been treated by several physicians without any permanent benefit. His symptoms led me to consider the case as one of 'regurgative disease,' so termed by Sir Henry Marsh.† Accordingly he was ordered to take systematic exercise, cool baths, and tonics in the form of elixir calisayæ and liquor potassii arsenitis, the latter in two-drop doses before meals. Three weeks later he reported a gain in

---

**Medical Press of Western New York*, February, 1886.

†Habershon, *Diseases of the Stomach*, 3d ed., p. 220.

weight of four pounds and symptoms somewhat improved. The improvement, however, was not permanent and he soon ceased taking exercises in the gymnasium, and, after several changes in medication with no benefit, that also was discontinued. He was able to walk about and do some work, but at times his trouble would be so bad as to render him unable to do anything.

"Was called to see him again on July 9th, and found him rather weaker than usual, he having been unable to retain much of anything for several days. From a careful study of his swallowing at that time I was led to the conclusion that the trouble must be in the esophagus and that, as the patient thought, the food was rejected before reaching the stomach. Anything that passed down into the stomach was retained and digested. It occurred to me that the trouble might be a spasmodic stricture of the esophagus. On the next day I took the patient to see Dr. Park in consultation. At this examination he passed a good-sized esophageal bougie into the stomach, without the slightest muscular resistance, which rather set aside the idea of a stricture. After further careful study of the case the diagnosis was made of diverticulum of the esophagus to the right side. The prognosis was accordingly unfavorable. Feeding for a time by means of the stomach tube was recommended, but was rejected by the patient. Seeing him several days later, he said he felt better since the examination, and had been able to retain nearly everything he took. Since then, however, he has been about as before, although at the present time his appearance is better than six months ago. I may add that his pulse is now steadily 90 and his temperature normal."

The subsequent history is one of continued difficulty in getting food into the stomach. Swallowing never has become normal, there having been always some stoppage of food above the cardiac orifice. Five years ago he was obliged to cut out solids, subsisting since on milk, soaked bread, cereals and eggs. Still he has been able to follow for years the occupation of candy salesman, as wholesaler to small stores, having his own route and using horse and wagon. He has lately given this up because of increasing difficulty of maintaining nourishment and the development of a positive degree of general muscular weakness. January 17, 1913, at the age of 48, he entered the Buffalo General Hospital.

Family history: Father living and well at 84; mother died at 76; three brothers died with scarlet fever; one sister living and well. There is no history of chronic disease or taint in family.

Personal history: Had scarlet fever when a child, but no other disease except the present ailment.

His habits have always been good; takes a glass of beer occasionally and smokes moderately; denies any venereal history.

Present appearance: He is somewhat emaciated, weighing 116 pounds; gait unsteady, with general deficiency of muscular power; voice hoarse and low-pitched. Sight is defective, requiring dark glasses to protect eyes from strong light. Temperature is normal, pulse 85.

Complaint: Difficulty in swallowing, which has persisted for thirty years and which for the past three years has been accompanied by an increasing sense of pressure and pain in the course of the esophagus. Muscular weakness. Frequent vomiting, sometimes of material taken a couple of days previously. Cough, not constant, but rather troublesome at times when eating, when he also is liable to "choke up" and have difficulty in breathing. Appetite is poor and bowels constipated.

Physical examination: Muscular atrophy is quite evident and seems general, especially marked in the small muscles of the hands. Reflexes generally exaggerated.

Lungs: Respiratory movement poor with diminished breathing. Marked depressions above both clavicles. Percussion resonance increased over entire right chest anteriorly, diminished in suprascapular regions and extending on both sides of spine to bases posteriorly. Breathing sounds vesicular, but diminished in volume. There are no rales to be heard.

Heart: Size not appreciably altered. Sounds are weak but well balanced. There are no murmurs. Blood pressure, 140.

Abdomen: Liver dullness from fourth interspace in nipple line to one-half inch below costal margin, where it is palpable. Spleen dullness normal. There are no palpable masses or tender areas. A few lymph nodes palpable in inguinal regions, none in cervical or axillary.

Blood: Hemoglobin, 80 per cent.; red cells, 4,340,000; leucocytes, 5,700.

Differential:

| | |
|---|---|
| Polymorphonuclears | 50% |
| Small lymphocytes | 33% |
| Large lymphocytes | 7% |
| Transitional forms | 8% |
| Basophiles | 2% |

Urine: Out of three analyses a very faint trace of albumin is reported once. Microscope shows a few leucocytes in each and occasional fine granular casts in one. Urine generally concentrated, no sugar, indican normal.

The Von Pirquet test is positive. (Jan. 25th.)

The Wassermann test is negative. (Jan. 30th.)

Sputum: (Jan. 20th.) Scanty; contains numerous diplococci, both intra and extra-cellular; short, thick bacilli, in chains of 4 to 8; no tubercle bacilli.

Muscular and nervous systems: (Dr. Putnam.) Muscular atrophy pronounced in small muscles of hands, and more marked on left side. Absence of power adduction of little fingers on both sides. Upper arm and shoulder group of muscles show good tone. Muscles of palate are weak, more marked on left side.

Reflexes of upper extremities are all exaggerated, also the knee jerks. Babinski, Kernig and Chadduck signs all negative.

LATERAL VIEW.

There is no ankle clonus. The condition is regarded as progressive muscular atrophy of the arm type (spinal).

Examination of eyes showed the conjunctivæ rough, and a superficial scar on left cornea. Temporal margins of discs are pale—probably atrophic. Pupillary reflexes active to both light and accommodation.

Inasmuch as a diagnosis of esophageal diverticulum had been made years earlier, chief interest centered in the upper digestive tract. The newer methods of diagnosis, unknown when the case was studied previously, gave reports as follows:

X-Ray examination: (Dr. Plummer.) Bismuth solution swallowed. Anterior view: Both lungs show dense areas. There is a distinct shadow along left of spine, probably esophagus with bismuth solution.

Lateral view: Bismuth solution has passed into the stomach. Esophagus shows distortions at two points, which may be caused by an irregular diverticulum, but seems more likely to be a tumor of mediastinal space involving wall of esophagus.

ANTERO-POSTERIOR VIEW.

In connection with the above opinion, repeated careful examinations excluded aortic aneurism.

Larynx and esophagus by direct examination: (Drs. Hinkel and Fairbairn). January 29th. The arytenoids are relaxed, slightly edematous in appearance and somewhat "clubbed." A slightly nodular, sessile tumor, about one-fourth inch in diameter, projects from the interarytenoid space above the plane of the vocal bands. Its color is the same as that of the laryngeal mucosa. Soft palate is relaxed and almost immovable with phonation. The left arytenoid and the left vocal band are immovable both with respiration and phonation. Vocal bands are pink but not swollen.

Esophagoscopy shows a greatly dilated esophagus without any diverticulum or adhesion being found. The fundus of the dilated portion contains semifluid accumulation which prevents a view of the cardiac orifice.

February 4th. After lavage of the dilated esophagus, examination with Bruning's esophagoscope shows enormous dilatation down to the cardia; musoca fairly normal; passed a small olivary bougie 18¾ inches from the teeth and through cardia with but little difficulty, but with moderate resistance each way.

February 6th. Passed a large olivary bougie well into the stomach. Laryngeal irritation and even dyspnea frequently attended the passage of instruments.

As the result of these observations the diagnosis of the esophageal condition as one of cardiospasm seems entirely proper. The accompanying muscular weakness and atrophy must be regarded as independent, at least as to origin, having developed later than the esophageal disorder; although the consequent disability has undoubtedly been aggravated by the lack of sufficient nourishment.

Treatment was then directed toward relief of the spasm of the cardia. The Sippy dilator was used by Dr. Eschelman, its introduction being usually preceded by the passage of a large olivary bougie. From February 14th until March 10th dilatation was practiced twice a week, or eight times, applying a pressure of from 150 to 200 m. m., as measured by the "Tycos" blood pressure gauge. Improvement followed the first dilatation and continued, so that after the eighth treatment the patient was allowed to rest from treatment until March 29, when, before leaving the hospital, another dilatation was employed. The nearly three weeks' interval without treatment showed no retrogression. During the period of treatment the patient gained about six pounds in weight.

Present condition of patient (November 1, 1913): He has been without treatment for seven months and at work at his former occupation for six and one-half months. He is able to eat an ordinary variety of food, including fruits and meat. Pain and pressure at meals is slight and of short duration, accompanying a sense of stoppage which always occurs when food or drink is swallowed. This sensation is temporary and disappears with "dropping of food into the stomach." Patient can always tell when the latter occurs. He thinks all food reaches the stomach by the time the meal is ended. He has gained eight pounds in weight. He is stronger in so far as better nutrition can contribute in his case, but the muscular atrophy is still evident and the actual muscular power is not much increased.

The cough and "choking up" in connection with eating have disappeared. His voice still has the same hoarse quality.

It is evident, therefore, that the spasm at the cardia has lessened very greatly under the treatment by dilitation; while the muscular condition remains stationary and justifies the diagnosis of progressive muscular atrophy.

---

RAPID CLINICAL METHOD FOR THE ESTIMATION OF UREA IN URINE. By E. K. Marshall, Jr. (*Journal of Biological Chemistry,* April, 1913. The method consists in incubating a portion of urine with an acqueous extract of soy bean flour, all the urea being thereby transformed into ammonium carbonate through the action of an enzyme existing in the soy bean. To prepare the extract, 25 gms. of soy bean powder are mixed with 250cc. of distilled water and allowed to stand an hour. 25cc. N/10 HCl are then added, allowing the mixture to stand a few minutes longer. This precipitates most of the protein, which is then removed by filtration. A few drops of toluene are added to the filtrate as a preservative. The urea determination is as follows: Two 5cc. portions of urine are measured into flasks of 200-300 cc. capacity and diluted with distilled water to 100-125 cc. 2 cc. of enzyme solution are added to one flask, a few drops of toluene to each, and the solution allowed to remain well stoppered at room temperature over night. The fluid in each flask is then titrated to a distinct pink color with N/10 HCl, using methyl orange as an indicator. The amount of HCl required for the urine and enzyme solution, less the amount used for the urine alone and the amount (which must have been previously detained) required to similarly titrate the enzyme solution corresponds to the urea present in the urine. 1cc. N/10 HCl corresponds to 0.6 gm. per liter of urea in the urine. The error of the method is under 2 per cent.

---

FISH DIET AND IODINE CONTENT OF THYROID. The observation that a fish diet increased the iodine content of a dog's thyroid was made some time ago. In the October number of the *Biochemical Journal,* A. J. Cameron states that definite evidence has been obtained that large quantities of iodine are constantly present in the thyroid in some species of salt-water fishes. The largest amount was found in the thyroid of scyllium canicula, and in one case much more iodine than in any thyroid previously reported upon. The research suggests that fish thyroid would be a valuable theraneutic agent, and that the iodine value of the diet plays an important part in determining that of the gland.

## Figures and Tables

**Figure f1:**
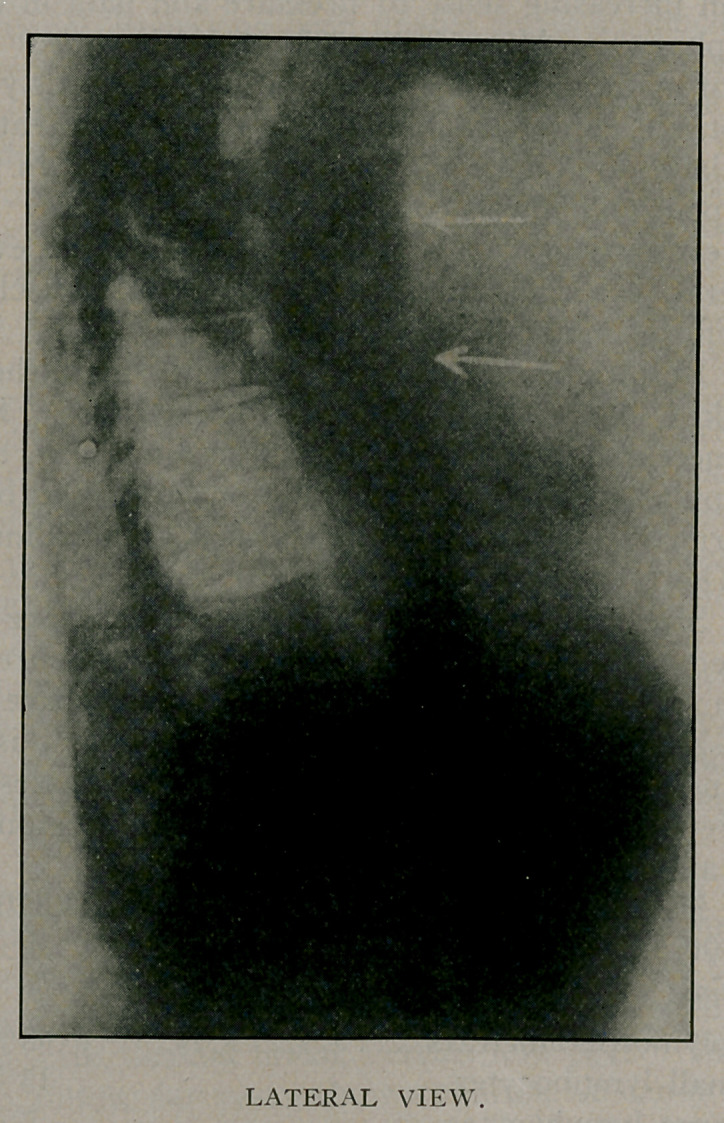


**Figure f2:**